# The Developmental Autism Early Screening (DAES): A Novel Test for Screening Autism Spectrum Disorder

**DOI:** 10.1007/s10803-023-06184-3

**Published:** 2023-12-18

**Authors:** Lara Cirnigliaro, Maria Stella Valle, Antonino Casabona, Martina Randazzo, Francesca La Bruna, Fabio Pettinato, Antonio Narzisi, Renata Rizzo, Rita Barone

**Affiliations:** 1 Child Neurology and Psychiatry Unit, Department of Clinical and Experimental Medicine, University of Catania, Policlinico Via Santa Sofia, 78, 95123 Catania, Italy; 2 Laboratory of Neuro-Biomechanics, Department of Biomedical and Biotechnological Sciences, School of Medicine, University of Catania, Catania, Italy; 3 IRCCS Stella Maris Foundation, Pisa, Italy; 4 Reseach Unit of Rare Diseases and Neurodevelopmental Disorders, Oasi Research Institute-IRCCS, Troina, Italy

**Keywords:** Autism spectrum disorder, Early diagnosis, Developmental profile, Griffiths III

## Abstract

This study was undertaken to set a novel developmental screening test for autism spectrum disorder (ASD) using the Griffiths Scales of Child Development (Griffith III) (Green et al., 2016; Stroud et al., 2016), in order to intercept the early atypical developmental patterns indicating ASD risk in the first 3 years of age. An observational and interactive ASD screener, the Developmental Autism Early Screening (DAES), was developed by detecting Griffiths III items differentiating toddlers with ASD risk from those with global developmental delay (DD) or neurotypical development. The DAES was validated with ASD-specific diagnostic instruments (ADOS-2) and the cut-off score based on sensitivity, specificity and positive predictive value that best differentiates between ASD and non-ASD children was identified. We enrolled a total sample of 297 subjects, including children at risk for ASD or DD and neurotypical children. At a cut-off score of 12.5, the DAES had a sensitivity of 93%, specificity of 98.4%, positive predictive value of 96.3% and negative predictive value of 96.9% for identifying children at risk for ASD from non-ASD participants (DD/neurotypical children). The DAES total score correlated significantly with the ADOS-2 calibrated severity scores (CSS) (R = 0.53, p < 0.001). Three ASD risk ranges were identified according to DAES total and ADOS-2 CSS: Little-to-no risk (CSS: 1–3, DAES: 1–7); Mild-to-moderate risk (CSS: 4–5, DAES: 8–14); Moderate-to-severe risk (CSS: 6–10, DAES ≥ 15). The DAES provides a direct approach based on developmental profiles to stratify risk for ASD in early childhood ensuring at risk children the most appropriate diagnostic procedures and targeted intervention.

**Supplementary Information:**

The online version contains supplementary material available at 10.1007/s10803-023-06184-3.

## Introduction

Autism spectrum disorder (ASD) is a neurodevelopmental disorder characterized by social communication impairment and restricted/repetitive behaviors and interests. The prevalence rate of ASD is 27.6 per 1.000 (one in 36) 8-year-old children in the United States (US). The average age of diagnosis is about 49 months but current estimates suggest that the median age of diagnosis is between 42 and 59 months in the US (Maenner et al., 2023). This is much later than possible even with only clinical evaluation and not biomarkers. In fact, there is much evidence that a sound diagnosis is possible and in fact practiced at 18 to 24 months (McCarty & Frye, 2020). According to a recent survey on ASD conducted in 14 European countries (ASDEU project network), the average age of access to diagnostic services in Europe is approximately 36.4 months and diagnoses occur between 36 and 42 months. However, the average age at which concerns were first raised about the child subsequently being diagnosed with ASD was 18.3 months, which suggests a need for earlier diagnosis (Bejarano-Martìn et al., 2020).

The development of autism-specific screening has facilitated the early identification of children at risk (Robins, 2008). This is consistent with the fact that evidence-based early interventions designed to harness experience-dependent neuroplasticity improve developmental trajectories in ASD (Dawson, 2008; Lord et al., 2015). The American Academy of Pediatrics (AAP) recommends screening all children for symptoms of ASD through a combination of developmental surveillance and standardized autism-specific screening tests at 18 and 24 months of age (Hyman et al., 2020; Johnson et al., 2007). The large majority of ASD screeners designed for caregivers are based on a conceptual analysis of early communication development and identification of “red flags” commonly described as early ASD indicators (Magàn-Maganto et al., 2017). The Modified Checklist for Autism in Toddlers, Revised with Follow-Up (M-CHAT-R/F), is one of the most studied and implemented ASD screening tool worldwide. It is a 2-stage parent-report screener developed to identify children from 16 to 30 months with ASD risk, in the general population (primary, level 1 screener) (Campbell et al., 2017; Marlow et al., 2019). Recently, Aishworiya et al (2023) performed a meta-analysis of the specific performances of M-CHAT-R/F that showed a pooled sensitivity of 82.5%, higher than expected for a good screening tool (70–80%). The probability of ASD diagnosis following M-CHAT-R/F positivity (pooled positive predictive values, PPV) was estimated at 51.2% [95% CI 43.0–59.5] in low-risk samples. This implies that a positive screening with the M-CHAT-R/F is predictive of an ASD diagnosis in approximately 50% of children. However, as previously recognized (Robins et al., 2014; Weitlauf et al., 2015), the pooled PPV of the M-CHAT-R/F for the presence of any developmental disorder rises to 89% (Aishworiya et al., 2023). It has been pointed out that several factors, such as low ASD prevalence rates and false responses due to lack of parental awareness of expected socio-communicative milestones, are associated with lower PPV regardless of screening sensitivity (McCarty & Frye, 2020). On the other hand, parents may over-report the presence of developmental abilities, leading to false negative screenings. Most studies examining screening tools for developmental disorders present a limitation in the assessment of negative predictive value (NPV), due to the financial costs involved in testing large numbers of negative screening children for final diagnosis and the long time period over which the disorder could potentially develop (Robins, 2020). Taking into account the above mentioned limitations, using a sequence of primary and secondary screening tools is important to maximize the predictive value of screening.

Level 2 (secondary) screeners aim to identify children at risk of ASD either because they are already under observation for developmental concerns, or because they failed Level 1 screening, or because they are siblings of children with ASD (Petrocchi et al., 2020). Level 2 interactive screeners need to be confrontational instruments where a trained professional interacts with the child providing a quantitative score and limiting the risk of subjective judgements. According to important criteria such as replication in multiple health-care settings and accuracy of classification, some level 2 screeners warrant consideration for clinical application such as the Screening Tool for Autism in Two-Year Olds: STAT (Stone et al., 2004); the Baby and Infant Screen for Children with aUtIsm Traits–Part 1: BISCUIT-Part 1 (Matson et al., 2010); the Autism Detection in Early Childhood: ADEC (Nah et al., 2014); the Systematic Observation of Red Flags: SORF (Dow et al., 2017) and the Rapid Interactive screening Test for Autism in Toddlers: RITA-T (Choueiri & Wagner, 2015). In particular, the STAT and the RITA-T represent the two secondary screening instruments with the most evidence, valid for children aged 36 months or less. Limitations of level 2 screeners are related to the performances in discriminating ASD from different neurodevelopmental disorders at certain ages, inadequacy of studied sample sizes, consistency issues, limited involvement of independent researchers, requirements for training in test administration and test administration time (Brewer et al., 2020; Norris & Lecavalier, 2010; Petrocchi et al., 2020).

Screening for normal development may unravel children with potential developmental delays (DD; APA, 2013) including delays in social-communication. In this respect, some instruments such as the Ages and Stages Questionnaire, have been integrated with the M-CHAT-R/F to combine screening for DD and ASD (Hardy et al., 2015).

Recently, the importance of studying developmental trajectories and, consequently, developing new tools to probe the atypical developmental trajectories of ASD in young children has been emerging. Developmental assessment of children is instrumental for understanding the child’s developmental level at the time of testing, revealing strengths and weakness in the different domains of learning. This is relevant in order to plan further investigations and/or referrals for appropriate therapeutic interventions. Systematic evaluation of early developmental profiles illustrated some weakness in language, social and communication skills in children with ASD when compared to peers with developmental and/or language delays (Barbaro & Dissanayake, 2012; Delehanty et al., 2018; Mitchell et al., 2011; Torrens & Ruiz, 2021).

The Griffiths Scales of Child Development 3rd Edition (Griffiths III) is the latest version of the Griffiths Developmental Scales, a tool validated for developmental assessment in children with ASD from birth to 6 years (Sandberg et al., 1993). Griffiths III was standardized in 2015 on a representative sample from the UK and Ireland, thereafter it was published and adapted for other population samples, with a normative age range between 1 and 72 months (Green et al., 2016; Stroud et al., 2016). Previous research embedded the use of Griffiths scales in describing distinct psychomotor profiles related to discrete diagnostic classes in pre-school aged children (Jansen et al., 2020; Li et al., 2020). A recent study aimed to compare the Griffiths III developmental profile of children with co-occurring ASD + DD with that of a group of children with DD but not ASD. The two diagnostic groups exhibited lower age equivalent scores with respect to their chronological age in all the considered developmental domains. However, unlike the group with DD having an uniform decrease of expected performances in all the domains, children with ASD + DD showed an uneven profile with relative failures in Language and Communication and Personal-Social–Emotional subscales (Taddei et al., 2023).

We aimed to timely understanding predictors of the atypical developmental trajectories associated with the wide phenotypic variability in ASD-risk children. Identifying specific risk signs in the period of maximum brain plasticity could facilitate an early therapeutic intervention and a more favourable outcome. The present study was undertaken for developing and preliminarily validating a novel observational and interactive level 2 ASD screener, the Developmental Autism Early Screening (DAES). The DAES was conceived from psychomotor developmental figures stemmed from the Griffiths III. In particular, we hypothesized that the Griffiths III might intercept the early recurrent atypical developmental patterns in children at ASD-risk in the first years of life. The DAES, based on Griffiths III, was designed to detect significant differences in the developmental patterns of children at risk of ASD, with developmental age 12 to 36 months, compared with children with developmental delay (DD) and typically developing (TD) children of the same developmental age. We report on the development, validity and discriminative properties of the DAES in differentiating young children at true risk of ASD from those with DD/neurotypical development. Based on the present study, we foresee that the DAES could complement current screeners and have the potential for widespread use due to its ease of administration and interpretation.

## Methods

### Study Design

We conducted an instrumental, quantitative and descriptive study divided into two phases: in phase I, the DAES was developed by clinicians experienced in assessing children with neurodevelopmental concerns. In phase II, the reliability and validity of the instrument were assessed.

#### DAES Development

The DAES has been developed by comparing Griffiths III scores obtained in children at risk of ASD, children at risk of DD and TD children. For this purpose, 78 subjects were consecutively recruited between January 2019 and June 2021, including 'clinically referred' children with expressed concerns for either ASD or DD and neurotypical children. Participants were assessed by clinical evaluation using DSM-5 criteria and the Griffiths III. They were matched for developmental age, as measured by the Griffith III A-subscale, in order to compare children with the same non-verbal cognitive level between groups (Table 1).

**Table 1:** Demographic features and developmental profiles (developmental ages) on the Griffiths III of participants recruited for test development

Participants	ASD	DD	CTRL	F2	p value
N. 78 (sex)	26 (M: 20)	26 (M: 22)	26 (M: 18)		
CA (months) (mean ± SD)	39.46 ± 12.48	34.07 ± 7.88	26.38 ± 7.48	12.288	**0.00002**
A-Scale					
DA (months) (Mean ± SD)	24.38 ± 5.80	24.5 ± 6.28	25.23 ± 5.87	0.152	0.8588
B-Scale					
DA (months) (mean ± SD)	14.61 ± 7.35	18.27 ± 7.47	25.46 ± 8.52	13.00	**0.00001**
C-Scale					
DA (months) (mean ± SD)	22.92 ± 7.50	23.19 ± 6.89	25.23 ± 7.03	0.809	0.4488
D-Scale					
DA (months) (Mean ± SD)	17.0 ± 6.21	22.15 ± 7.20	26.53 ± 6.64	12.345	**0.00002**
E-Scale					
DA (months) (mean ± SD)	25.73 ± 6.60	26.07 ± 7.07	26 ± 7.08	0.017	0.9822

Bold indicates statistically significant values (p ≤ 0.05)

*CA* chronological age, *DA* developmental age, *M* males, *ASD* autism spectrum disorder, *DD* global developmental delay, *CTRL* neurotypical children

The inclusion criteria were: A-subscale developmental age (DA) of 12–36 months; clinical referral for ASD-risk or DD-risk (first and second group, respectively). TD children were defined as having General Development Quotient ≥ 90 at Griffiths III (CTRL group). They were healthy children prospectively controlled for transient neonatal jaundice, suspected maternal infection, or late-preterm born infants (gestational age > 34 weeks) with no signs of neonatal distress.

Exclusion criteria included bilingualism; syndromes or genetic abnormalities; diagnosis of other neurological disorders (i.e. epilepsy, hearing and visual defects). A best-estimate clinical diagnosis of ASD or non-ASD was made by experienced clinicians using all available information and all testing measures, including the Autism Diagnostic Interview-Revised (ADI-R) and the Autism Diagnostic Observation Schedule, 2nd edition (ADOS-2) when clinically indicated.

#### DAES Validation

**Fig. 1:**
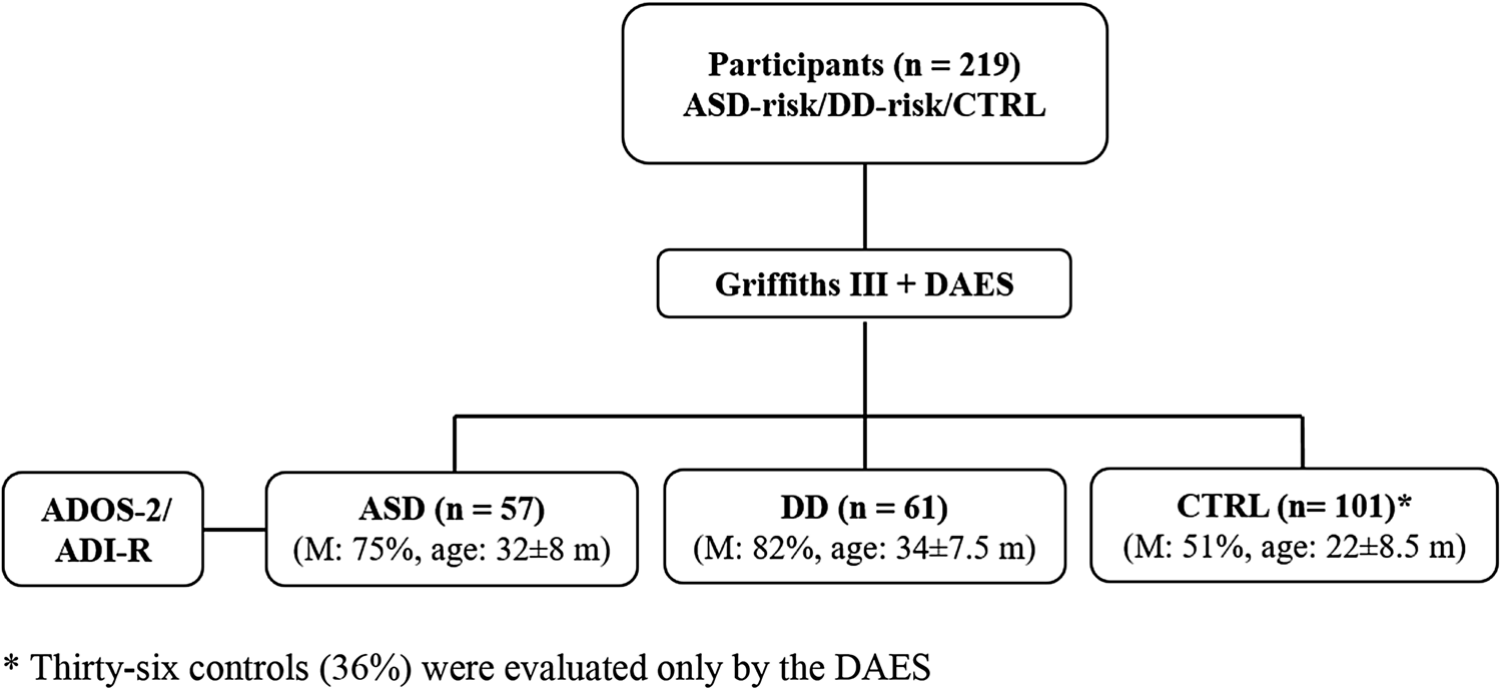
Participant flowchart for DAES validation. *ASD* Autism Spectrum Disorder, *DD* Global Developmetal Delay, *CTRL* control, *ADOS-2* Autism Diagnostic Observation Schedule, *ADI-R* Autism Diagnostic Interview-Revised, *M* males, *m* months

In phase two, we randomly recruited three experimental groups: two groups of 'clinically referred' children with parental or professional concerns about ASD and/or DD, and one group without developmental concerns (CTRL). The CTRL group consisted of healthy toddlers prospectively screened for transient neonatal jaundice, suspected maternal infection or preterm birth without signs of neonatal distress (Apgar scores: 9/10). Participants were recruited at the study site between July 2021 and June 2022 using the same inclusion and exclusion criteria as in the previous analysis. They had a chronological age ranging from 18 to 48 months with a Griffith III A-Subscale DA equal to 12–36 months. Participants were assessed by clinical evaluation and by the Griffiths III. The DAES was administered at the time of clinical evaluation by two research assistants at the referral university hospital for Child Neuropsychiatry where this study was based. The Cohen κ statistic was calculated for each rater and varied between 0.7 and 1, indicating good to excellent agreement. Two senior board-certified researchers blind to the DAES scores generated final diagnoses independently of the DAES based on a full assessment (history, observation, and all testing measures) (Fig. 1). Written informed consent from both parents was acquired before the beginning of the study. The current study was part of an overall larger study aimed at identifying markers, predictors and developmental trajectories of ASD. The larger overall study was approved by the local ethics committee at the University Hospital Referral Centre for ASD with number n° 759. All procedures performed in the present study were in accordance with the 1964 Declaration of Helsinki and its later amendments (2013).

### Measures

Participants were assessed using the Griffiths III that provides an overall measure of child psychomotor development across five subscales. (Green et al., 2016). Subscale A (Foundations of Learning) assesses the ability of learning (including attention, problem-solving abilities, sequential reasoning, processing speed, visuospatial skills and memory); subscale B (Language and Communication) evaluates the development of both receptive and expressive language and social communication abilities; subscale C (Eye and Hand Coordination) assesses visual perception and fine motor skills; subscale D (Personal-Social-Emotional) evaluates child’s ability to adapt, personal autonomy and early social and emotional development through items measuring imitation, joint attention, emotional recognition and empathy; subscale E (Gross Motor domains) refers to the child’s early development of postural control, gross body coordination, balance and visual-spatial coordination. Each item is scored as a pass or a fail, + 1 or 0 respectively. Subscale raw scores and general development raw scores are calculated to determine the Developmental Age, Scaled Score and Development Quotient, according to the norm tables.

DSM-5 criteria defined by the American Psychiatric Association were used for ASD and DD diagnosis (American Psychiatric Association, 2013). Symptoms of ASD were established using the gold-standard tools for ASD diagnosis: Autism Diagnostic Interview-Revised (ADI-R) (Lord et al., 1994) and the Autism Diagnostic Observation Schedule, 2nd edition (ADOS-2) (Lord et al., 2012) The ADI-R is a structured interview to the parents of children referred for evaluation of possible ASD. The ADOS-2 is a semi-structured, standardized assessment of core deficits in ASD. It contains five modules that are differentiated by children’s developmental and language levels. In the present study participants with ASD risk completed the Toddler Module (designed specifically for children 12–30 months old with limited language), the Module 1 (used for children aged from 31 months who do not consistently use phrase speech) or the Module 2 in a minority of children using phrase speech, but who were not verbally fluent. To allow comparisons among different modules, ADOS-2 scores (total score, Social Affect, SA, and Restricted and Repetitive Behaviour, RRB, scores) were converted to respective calibrated severity scores (CSS 1–10 indicating absence to severe autism) (Esler et al., 2015; Gotham et al., 2009; Hus et al., 2014). Raw SA domain scores were standardized using the same 10-point severity rating scale as total-raw scores. Instead, raw RRB domain scores were standardized in CSS values from 5 to 10, due to the limited range of the RRB raw total (Hus et al., 2014). Thus, a RRB CSS of 5 represents raw scores in the mild-to-moderate concern range.

### Statistical Analyses

In the first phase of tool development, we initially considered and compared equivalent mental/developmental ages (months) as means (M) and standard deviations (SD) on the Griffiths III in the three groups (ASD, DD and CTRL). The one-way ANOVA statistical test was applied for each Griffiths III subscale (A-E) in order to find out possible significant differences among groups.

A multiple comparison test was then performed (Tukey HSD test) to understand how the impact of each group could determine the statistical differences found.

Based on previous analyses, we continued focusing on the Griffiths III B and D-subscales, in the first 3 years of age, as language and social and emotional skills are mostly impaired in ASD. For tool development all items of B- and D-subscales were considered and grouped by year of age and by constructs. The scores obtained for each single item in the three different groups were analysed by the Pearson’s Chi-square independence test. The Chi-square test was applied to assess which items showed significant differences over the three groups and between each pair of groups: ASD/CTRL, ASD/DD and DD/CTRL group. Items significantly different between ASD and DD or CTRL groups, and therefore most predictive of ASD risk, were used to develop the instrument (DAES).

In the second phase (tool validation), the one-way ANOVA test with pairwise comparisons, based on Bonferroni correction, was applied to compare chronological age (CA) and developmental age (DA) among participants recruited for the test validation and including three groups (ASD, DD and CTRL).

Receiver operating characteristic (ROC) curve analysis was performed to validate the capability of the DAES total score in discriminating ASD children vs children with typical development (CTRL) and ASD children vs children with DD. Sensitivity, specificity, positive predictive value (PPV) and negative predictive value (NPV) of the DAES total score were calculated and the cut-off values, with the optimal sensitivity and specificity, were determined by using Youden’s J index and Euclidean distance (Akobeng, 2007; Kruizinga et al., 2014).

Linear correlations between DAES total score and ADOS-2 total CSS, SA CSS and RRB CSS, were calculated using the Spearman’s rank correlation and the strength of correlation was assessed by Spearman’s Rho coefficient (R).

The statistical significance level α was established at 0.05. All statistical tests were performed by using SPSS version 27 (SPSS, Inc., Chicago, IL, USA, IBM, Somers, NY, USA).

## Results

### DAES Development

For test development, the three groups (ASD, DD, CTRL) were compared for significant differences on each Griffith III subscale. Participants were matched on their developmental age (DA) on subscale A, expression of the learning base: mean participant DA was not significantly different across the three study groups (F = 0.152; p = 0.858). It was thought that this might give more weight to any differential elements in the achievement of the specific items provided in the other scales. A significant difference among groups was found for the B (F = 13; p = 0.00001) and D subscale DA means (F = 12.3; p = 0.00002). No differences were found in the remaining Griffith III subscales (Table 1).

A multiple comparison test (Tukey HSD test) was then carried out to understand how the impact of each group could determine the statistical differences found. DA significant differences were found between the ASD group and the CTRL group on the B subscale (p = 0.00001) and between the ASD group and both the DD (p = 0.029) and CTRL (p = 0.0001) groups on the D subscale.

From this initial survey, we deduced that the Griffiths III B and D subscales were the most sensitive in capturing differences between groups. We therefore focused on each individual item of the B and D subscale constructs that differed significantly in the comparisons between ASD and the remaining two groups, and this analysis was carried out for each year in the first 3 years of life (Table 2a, b). The items significantly different between groups representing the most predictive ones for ASD risk were used to develop the DAES (Table S1, Supplementary material).

**Table 2:** Griffiths III items with significant differences among groups (ASD/DD/CTRL) in subscales B (a) and D (b) constructs for year (Pearson’s Chi-square independence test)

(a)							
1st year	Listening, attention		Communicative intent		Preverbal expressive communication	Preverbal receptive language development	
	B1	B6	B13	B14	B11	B10	B16
3 groups							
χ^2^	8.432	14.319	9.043	7.8	8.177	7.8	8.177
*p*	**0.015**	** < 0.001**	**0.011**	**0.02**	**0.017**	**0.02**	**0.017**
ASD/DD							
χ^2^	4.333	6.584	5.318	2.364	0.591	2.364	3.519
*p*	**0.037**	**0.01**	**0.021**	0.124	0.442	0.124	0.061
ASD/CTRL							
χ^2^	4.333	9.455	5.318	6.783	5.532	6.783	6.933
*p*	**0.037**	**0.002**	**0.021**	**0.009**	**0.019**	**0.009**	**0.008**
DD/CTRL							
χ^2^	0	1.02	0	2.08	3.184	2.08	0.754
*p*	0	0.313	1	0.149	0.074	0.149	0.385

2nd year	Listening, attention	Expressive language development			Receptive language development	
	B2	B9	B10	B13	B3	B14
3 groups						
χ^2^	5.318	9.743	19.808	20.748	6.797	9.77
*p*	**0.021**	**0.008**	** < 0.001**	** < 0.001**	**0.033**	**0.008**
ASD/DD						
χ^2^	0.843	2.342	4.282	4.328	1.949	1.444
*p*	0.358	0.126	**0.039**	**0.04**	0.163	0.229
ASD/CTRL						
χ^2^	5.65	9.665	19.692	20.17	6.718	9.433
*p*	**0.017**	**0.002**	** < 0.001**	** < 0.001**	**0.01**	**0.002**
DD/CTRL						
χ^2^	2.364	2.769	6.718	7.076	1.564	3.775
*p*	0.124	0.096	**0.01**	**0.008**	0.211	0.052

(b)*							
1st year	Personal	Social					Emotional
	D13	D3	D4	D14	D15	D18	D16
3 groups							
χ^2^	8.488	9.797	9.73	21.589	9.797	14.585	7.8
*p*	**0.014**	**0.007**	**0.008**	** < 0.001**	**0.007**	** < 0.001**	**0.02**
ASD/DD							
χ^2^	1.981	3.359	4.127	0	3.359	5.026	2.364
*p*	0.159	0.067	**0.042**	**0.002**	0.067	**0.025**	0.124
ASD/CTRL							
χ^2^	8.089	8.089	6.786	15.6	8.089	12.381	6.783
*p*	**0.004**	**0.004**	**0.009**	** < 0.001**	**0.004**	** < 0.001**	**0.009**
DD/CTRL							
χ^2^	3.184	2.08	1.02	2.08	2.08	3.184	2.08
*p*	0.074	0.149	0.313	0.149	0.149	0.074	0.149

2nd year	Personal					Social				Emotional	
	D4	D8	D10	D14	D15	D2	D3	D7	D9	D2	D13
3 groups											
χ^2^	3.914	21.775	6.424	11.209	6.413	10.079	10.079	7.682	8.203	13.689	9.742
*p*	**0.048**	** < 0.001**	**0.040**	**0.004**	**0.041**	**0.006**	**0.006**	**0.021**	**0.017**	** < 0.001**	**0.008**
ASD/DD											
χ^2^	0.843	9.321	0.361	1.997	3.059	4.591	4.591	2.882	1.359	7.589	0.719
*p*	0.358	**0.002**	0.548	0.158	0.08	**0.032**	**0.032**	0.09	0.244	**0.006**	0.397
ASD/CTRL											
χ^2^	3.9	20.17	5.65	11.143	4.282	8.308	6.564	6.584	8.308	9.774	9.774
*p*	**0.048**	** < 0.001**	**0.017**	** < 0.001**	**0.039**	**0.004**	**0.004**	**0.01**	**0.004**	**0.002**	**0.002**
DD/CTRL											
χ^2^	1.209	2.882	3.359	2.882	0.115	0.754	0.754	1.083	3.359	0.221	2.882
*p*	0.271	0.09	0.067	0.09	0.734	0.385	0.385	0.298	0.067	0.638	0.09

3rd year	Personal				Social	Emotional
	D1	D2	D4	D7	D8	D5
3 groups						
χ^2^	15.023	19.343	11.361	15.631	12.057	6.413
*p*	** < 0.001**	** < 0.001**	**0.003**	** < 0.001**	**0.002**	**0.041**
ASD/DD						
χ^2^	1.879	6.799	2.877	3.972	2.722	1.089
*p*	0.17	**0.009**	0.09	**0.046**	0.099	0.297
ASD/CTRL						
χ^2^	12.92	19.461	10.909	15.084	11.879	4.713
*p*	** < 0.001**	** < 0.001**	** < 0.001**	** < 0.001**	** < 0.001**	**0.03**
DD/CTRL						
χ^2^	4.105	3.775	3.586	5.103	1.396	1.513
*p*	0.128	0.052	0.058	**0.024**	0.237	0.219

*Significant differences were not evident in the third year of age in B-subscale constructs

Bold indicates statistically significant values (*p* ≤ 0.05)

*ASD* autism spectrum disorder, *DD* global developmental delay, *CTRL* neurotypical control group

As an example, among the skills expected in the first year of age in the area of listening and attention, a significant difference between the three groups was found in items B1 (makes eye contact with the speaker) and B6 (responds when called—gets the child's attention in some way). These skills basically reflect the child's ability to establish an interaction with the interlocutor, an indispensable prerequisite for the acquisition of further communicative skills. Group comparison analyses using Pearson’s chi-squared test for independence revealed that children with ASD significantly failed both items B1 and B6 when compared with either the DD or CTRL groups (Table 2a).

As aforementioned, the items for years one to three, representing the most predictive for ASD risk, were added to figure the novel screening tool based on differences in early developmental profiles measured on the Griffith III: Developmental Autism Early Screening (DAES).

#### DAES Description and Administration

The DAES is a 36-items observational and interactive measure, developed by selecting those we found to represent the most predictive Griffiths III items for ASD risk in the first 3 years of age. Items are organized according to the Griffiths III specific constructs of B- (Language and Communication) and D- (Personal-Social-Emotional) subscales, that are expected particularly impaired in children with ASD. In the DAES, items probe the following areas: listening, attention and communicative intent, expressive communication, receptive language development, social-emotional reciprocity, social communication and interaction, play, social cognition, joint attention, self-awareness, emotional understanding and expression and empathy. The tool is administered in approximately 20 min, starting with the first items (starting points) regardless of the child's age. According to Griffith III, the discontinue administration rule is established after six consecutive items not passed within each subscale. No specific training is required for users trained in the use of the Griffith III. The standardised administration of items may refer to the Griffith III manual (Table S1, Supplementary Material).

#### DAES Scoring

Based on statistical analyses, we assigned a score of + 2 to the items that were significantly different in the ASD children compared to both the DD and CTRL groups, and a score of + 1 to the items that were significantly different in the ASD children compared to the CTRL group.

For each item, 0 represents the skill being expressed. Therefore, if the child fails the item (skill not expressed), a score of + 1/ + 2 is assigned during the observation, so that a higher total score indicates a higher risk of ASD. For example, if the child fails items B1.1 or B1.6 (significantly different in ASD children compared to both CTRL and DD groups), a score of + 2 is assigned, whereas if the child fails items B1.10 and B1.11 (significantly different in ASD group compared to CTRL group), a score of + 1 is assigned. If the child passes the items, a score of 0 is assigned.

### DAES Validation Analyses

For preliminary validation analyses, the DAES was administered to 219 children recruited over a 12-month period (ASD/DD risk and CTRL). Mean total DAES scores were higher in the ASD risk group (19 ± 5.4) than in the DD risk (6 ± 3.7) and CTRL (4 ± 3.5) groups.

According to DSM-5 criteria, Griffiths III, ADI-R and ADOS-2 scores, 57 children were diagnosed with ASD and 61 children were diagnosed with DD. 101 children were in the CTRL group (Fig. 1).

TD children (CTRL group) were significantly younger in comparison with ASD (p < 0.001) and DD participants (p < 0.001). DA (months) on Griffith III A subscale, was computed as measure of non-verbal mental development. DA was not significantly different when comparing ASD (20 ± 5.8 months) with CTRL groups (20 ± 8.4 months) (p = 1), considering the younger chronological age of the CTRL. DA was significantly lower in children with ASD than in children with DD (24 ± 6.3 months) (p = 0.005), indicating that, on average, participants with ASD were developmentally delayed. In fact, a minority of the ASD group in the study (20%) had no difference in DA/CA.

#### Assessment of the Diagnostic Accuracy of the DAES

**Fig. 2:**
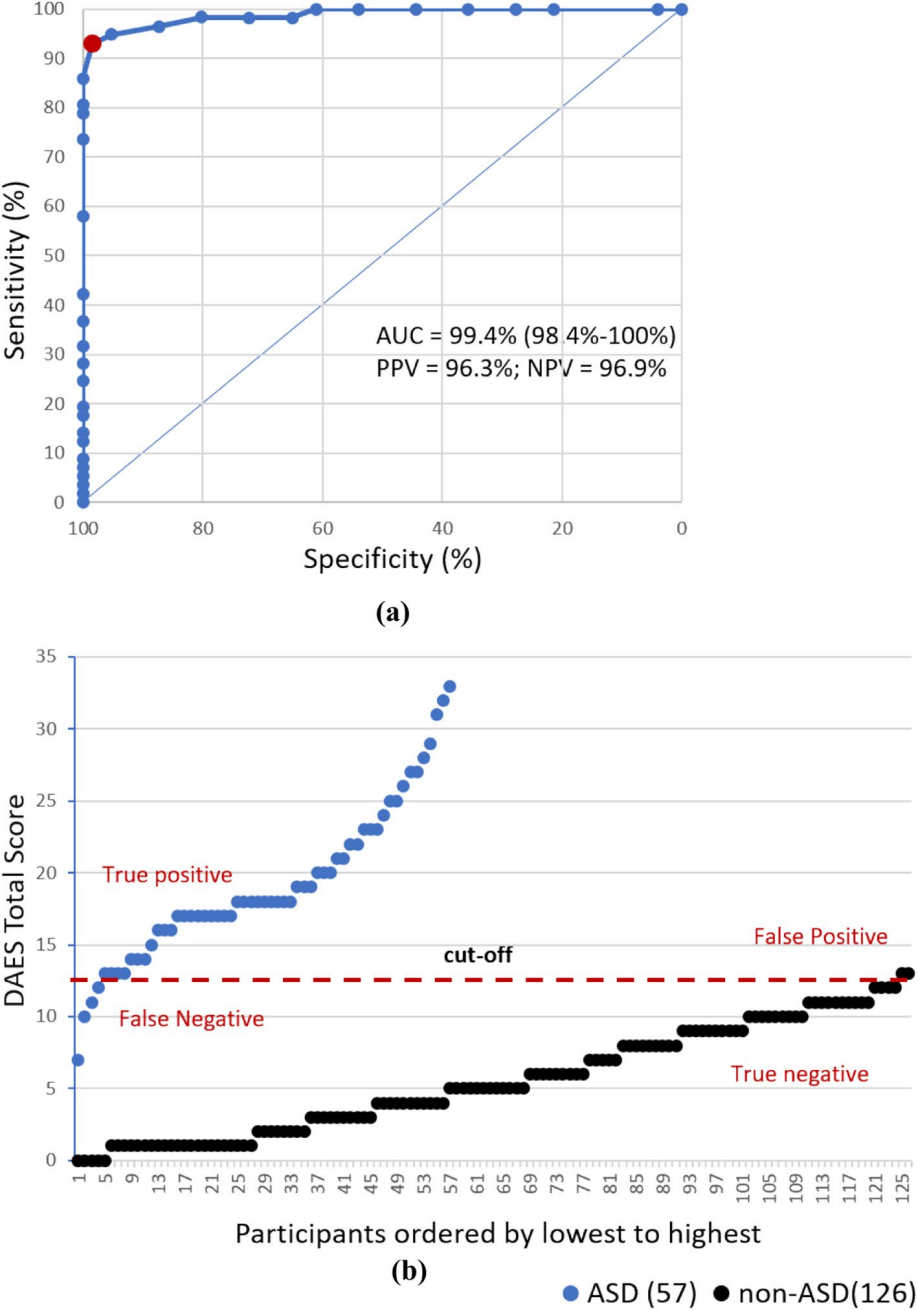
**a** ROC curve of DAES total score to evaluate sensitivity and specificity in separating ASD (n = 57) from non-ASD groups (DD + CTRL, n = 126). DAES total score cut-off of 12.5 (red circle) shows higher optimal sensitivity and specificity based on Youden’s J index and Euclidean distance. **b** Distribution of DAES total score for each participant of ASD (n = 57) and non-ASD groups (DD + CTRL, n = 126). With a cut-off value of 12.5, a total of 4 out of 57 were False Negative and a total of 2 out of 126 were false positive. thus, 53 were true positive and 124 were true negative

Considering the clinical diagnosis and the DAES total score, ROC curve analyses were performed to validate the predictive performance of DAES scores by determining its optimal cut-off score in discriminating ASD group from non-ASD participants (DD/CTRL groups). For these analyses, we included 65 TD children assessed by Griffiths III and DAES from the CTRL group (n = 101), in order to make the compared samples numerically more homogeneous. We found the area under the curve (AUC) of DAES Total Score was 0.994 (95% CI 0.98–1), thus supporting the DAES capacity in classifying the ASD group from the non-ASD group (Fig. 2a). In this application, the DAES Total Score cut-off of 12.5 showed higher optimal sensitivity (93%) and specificity (98.4%), with a PPV of 96.3% and NPV of 96.9% (Table 3).

**Table 3:** Sensitivity, specificity and other associated parameters of the ROC curve in separating ASD (n = 57) from non-ASD (DD + CTRL, n = 126) groups for different cut-off scores

Cut-off	Sensitivity (%)	Specificity (%)	False negative	False positive	True positive	True negative	PPV (%)	NPV (%)	Youden index	Euclidean distance
0.5	100.0	4.0	0	121	57	5	32.0	100.0	0.040	0.960
1.5	100.0	21.4	0	99	57	27	36.5	100.0	0.214	0.786
2.5	100.0	27.8	0	91	57	35	38.5	100.0	0.278	0.722
3.5	100.0	35.7	0	81	57	45	41.3	100.0	0.357	0.643
4.5	100.0	44.4	0	70	57	56	44.9	100.0	0.444	0.556
5.5	100.0	54.0	0	58	57	68	49.6	100.0	0.540	0.460
6.5	100.0	61.1	0	49	57	77	53.8	100.0	0.611	0.389
7.5	98.2	65.1	1	44	56	82	56.0	98.8	0.633	0.349
8.5	98.2	72.2	1	35	56	91	61.5	98.9	0.704	0.279
9.5	98.2	80.2	1	25	56	101	69.2	99.0	0.784	0.199
10.5	96.5	87.3	2	16	55	110	77.5	98.2	0.838	0.132
11.5	94.7	95.2	3	6	54	120	89.9	97.5	0.899	0.072
**12.5**	**93.0**	**98.4**	**4**	**2**	**53**	**124**	**96.3**	**96.9**	**0.914**	**0.072**
13.5	86.0	100.0	8	0	49	126	100.0	94.0	0.860	0.140
14.5	80.7	100.0	11	0	46	126	100.0	92.0	0.807	0.193
15.5	78.9	100.0	12	0	45	126	100.0	91.3	0.789	0.211
16.5	73.7	100.0	15	0	42	126	100.0	89.4	0.737	0.263
17.5	57.9	100.0	24	0	33	126	100.0	84.0	0.579	0.421
18.5	42.1	100.0	33	0	24	126	100.0	79.2	0.421	0.579
19.5	36.8	100.0	36	0	21	126	100.0	77.8	0.368	0.632
20.5	31.6	100.0	39	0	18	126	100.0	76.4	0.316	0.684
21.5	28.1	100.0	41	0	16	126	100.0	75.5	0.281	0.719
22.5	24.6	100.0	43	0	14	126	100.0	74.6	0.246	0.754
23.5	19.3	100.0	46	0	11	126	100.0	73.3	0.193	0.807
24.5	17.5	100.0	47	0	10	126	100.0	72.8	0.175	0.825
25.5	14.0	100.0	49	0	8	126	100.0	72.0	0.140	0.860
26.5	12.3	100.0	50	0	7	126	100.0	71.6	0.123	0.877
27.5	8.8	100.0	52	0	5	126	100.0	70.8	0.088	0.912
28.5	7.0	100.0	53	0	4	126	100.0	70.4	0.070	0.930
30	5.3	100.0	54	0	3	126	100.0	70.0	0.053	0.947
31.5	3.5	100.0	55	0	2	126	100.0	69.6	0.035	0.965
32.5	1.8	100.0	56	0	1	126	100.0	69.2	0.018	0.982

Bold indicates higher optimal sensitivity and specificity, with a PPV of 96.3% and NPV of 96.9% at a DAES total score cut-off of 12.5, determined by using Youden’s J index and Euclidean distance

*PPV* positive predictive value; *NPV* negative predictive value

A scatter plot with the distribution of the DAES total scores for each participant in the two groups (ASD/non-ASD) showed a total of 4 out of 57 false negative and a total of 2 out of 126 false positive; thus, 53 were true positive and 124 were true negative (Fig. 2b).

The ROC curve calculated for classifying ASD group with respect to CTRL group indicated a DAES cut-off score of 11.5 showing higher optimal sensitivity (94.7%) and specificity (100%), with a PPV of 100% and NPV of 95.6% (Fig. S1A–D). A scatter plot with the distribution of the DAES total scores for each participant in the two groups (ASD/CTRL) showed a total of 3 out of 57 false negative and a total of 0 out of 65 false positive; thus, 54 were true positive and 65 were true negative (Fig. S1A–D). An additional ROC curve was calculated for comparing the ASD group with the whole group of children with typical development (n = 101). This sample included also 36 children not assessed by Griffiths III. The DAES cut-off score of 11.5 showed higher optimal sensitivity (94.7%) and specificity (100%), with a PPV of 100% and NPV of 97%. A scatter plot with the distribution of the DAES total scores for each participant in the two groups (ASD/CTRL) showed a total of 3 out of 57 false negative and a total of 0 out of 101 false positive. These results are comparable to the previous ones, in which 65 children in the CTRL group were considered.

Then we computed the DAES performance in classifying correctly between ASD and DD. In this context, the DAES cut-off score of 12.5 showed sensitivity (93%) and specificity (96.7%), with a PPV of 96.3% and NPV of 93.7% (Fig. S1A–D). The scatter plot distribution of the DAES total scores for each participant in the two groups (ASD/DD) showed a total of 4 out of 57 false negative (53 were true positive) and a total of 2 out of 61 false positive (59 were true negative) (Fig. S1A–D).

#### Relationships Among the Different Measures

**Fig. 3:**
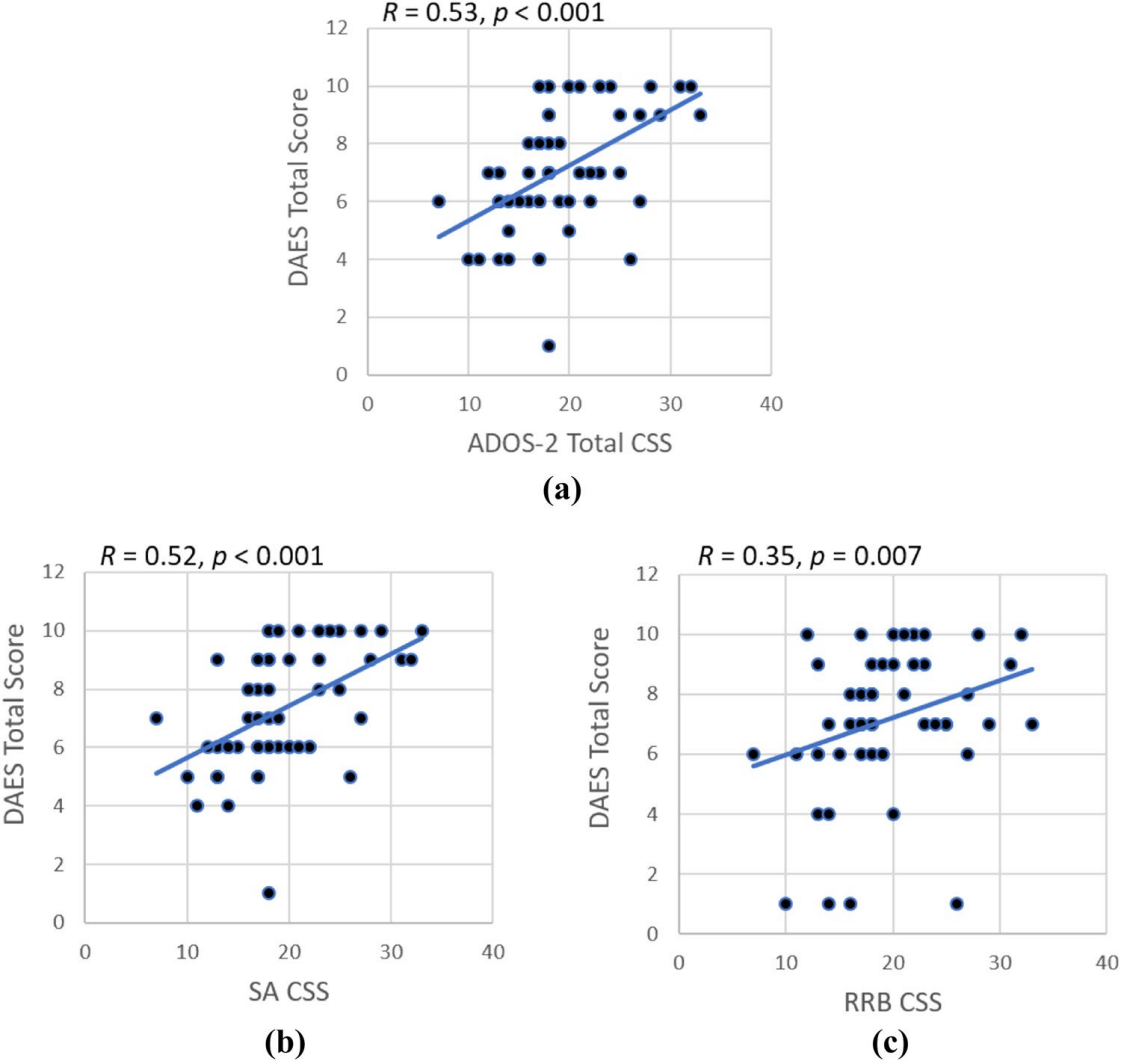
Linear correlations between DAES total score and **a** ADOS-2 total CSS (R = 0.53, p < 0.001), **b** SA CSS (R = 0.52, p < 0.001), and **c** RRB CSS (R = 0.35, p = 0.007), calculated using the Spearman’s rank correlation. The strength of correlation was assessed by Spearman’s Rho coefficient (R) and the level of statistical significance was set at p < 0.05

Linear regression was used to assess correlations between DAES score and ADOS-2. To allow comparisons between different modules, ADOS-2 CSS were used for correlation analyses. The DAES total score was found to be significantly correlated with the ADOS-2 total, SA and RRB CSS (Fig. 3a–c), which provide a measure of ASD symptom severity.

Three risk ranges of DAES total score were identified in the ASD group according to the ADOS-2 total CSS: Little-to-no risk (CSS: 1–3, DAES total score: 1–7); Mild-to-moderate risk (CSS: 4–5, DAES total score: 8–14); Moderate-to-severe risk (CSS: 6–10, DAES total score ≥ 15) (Fig. S2).

## Discussion

We report a new level 2 ASD screening tool based on developmental profiles obtained through a systematic analysis of all Griffiths III items in the first 3 years of age in young children at risk of ASD, at risk of DD and in TD children. Previous studies have highlighted the importance of assessing neurodevelopment in children with ASD using the Griffiths Scales in order to provide more targeted learning strategies. In fact, the Griffiths scales are increasingly being used as part of test batteries to establish baselines, aid diagnosis and monitor the development of children with a range of developmental disorders, including ASD (Jansen et al., 2020; Li et al., 2020; Taddei et al., 2023). In this regard, peculiarities in longitudinal assessment of psychomotor development using the Griffiths scales were searched for to assess ASD risk and symptom severity at diagnosis. Children with ASD showed lower scores of General Quotient and all sub-quotients measured on the Griffiths Mental Development Scale (GMDS) over time, with the exception of the performance sub-quotient. Interestingly, three sub-quotients (Personal-Social, Hearing and Language and Practical Reasoning) were associated with the symptom severity of ASD at the time of diagnosis (Pino et al., 2022). Moreover, Li et al. (2020) using the Chinese version of the GMDS explored the relationships among developmental levels and ASD severity, sex and age of the child at ASD diagnosis. Scores of sub-quotients were significantly lower in children with more severe ASD. The performance sub-quotient was significantly low in a higher proportion of girls than boys while motor abilities measured on the locomotor subscale decreased with age at diagnosis.

Limitations of current level 2 screeners include difficulties in the exploration of potentially discriminating items unravelling the developmental trajectories in ASD children. This, in turn, is functional for discriminating ASD from different neurodevelopmental disorders at certain ages. Thus, exploration of potentially discriminating items at the target age is particularly envisaged. To date, little is known on the potential application of the Griffiths III for early identification of children with ASD compared with both neurotypical children and children with developmental disorders different from ASD. Assuming that some peculiarities of the development of children with ASD can orient towards an early diagnosis of this condition, we sought a new level 2 screening tool through a systematic analysis of all Griffiths III items in the first 3 years of age in order to differentiate children with ASD risk from children with DD and those with typical development.

According with previous studies, we proved that B- and D-subscales are more sensitive in intercepting differences between the groups (Jansen et al., 2020; Li et al., 2020; Pino et al., 2022; Taddei et al., 2023). In particular, we pursued to identify the items of Griffiths III B- and D-subscales which showed a significant difference among the three groups (ASD, DD and CTRL) in the first 3 years of life.

In the first year ASD children showed more difficulties in acquiring communication skills required for age, compared to DD children. Significant differences among groups were found in the B-subscale constructs “listening and attention” and “intentional communication”, focused on the ability of children to build dyadic interactions. Children with ASD and DD specifically lacked social bases of language (i.e. use of gestures, facial expressions, joint attention), with respect to children with DD in the items of the first year of life. This is consistent with consolidated knowledge showing that children in the DD group may increasingly attend to the social world through more advanced joint attention skills, which, in turn, leads to better responsiveness to language (Barbaro & Dissanayake, 2012).

In the second year of age, the children with ASD showed greater deficits in the subscale B construct 'expressive language development' than the DD children, whereas these differences were no longer evident in the third year of age. In particular, in children with ASD, significant language development can occur between 24 and 48 months of age. Therefore, the level of language development at 48 months may predict language outcome in ASD (Brignell et al., 2018).

With regard to D-subscale constructs, in the first year of life, ASD children, compared to DD children, showed more deficits in acquiring skills of “social” construct, such as early referential understanding and joint attention. The analysis of the data in the second and third years highlighted a progressive divergence between the group of children with autism and the other two groups in the D-subscale constructs “personal” and “social-emotional”. In particular children with ASD failed in imitation, dyadic interactions and self-processing skills (self-concept and self-awareness) compared to children with DD.

Altogether these results are consistent with recent studies focused on the assessment of ASD children compared with DD children through Griffiths scales, that highlighted a more significant impairment in the personal and social domains in ASD children (Taddei et al., 2023; Wang et al., 2021). The presence of communicative and social deficits in children with ASD confirms that the core features of ASD translate into a specific profile of early psychomotor functioning. The DAES, developed by analysing the most predictive Griffiths III items for ASD risk, may assist in the identification of young children at risk of ASD in relation to DD, in order to target intervention prior to formal diagnosis.

For this purpose, we assessed the DAES ability to differentiate children with ASD from those non-ASD including children with DD or with neurotypical development in a validation sample. Overall, we found a unique DAES total score cut-off of 12.5 enabling differentiation between ASD group from a non-ASD group (DD/CTRL), in the first 3 years of life, with high accuracy, showing a sensitivity of 93% and a specificity of 98.4% (PPV: 96.3%, NPV: 96.9%).

Notably, the DAES correlated positively with the ADOS-2 CSS scores and with its diagnostic assignment by clinicians who were blinded to the DAES test results. Because of its significant correlation with ADOS-2 scores, the DAES has its value in stratifying those children at risk for ASD. In fact, following the overall ADOS-CSS risk stratification, we further identified an ASD risk score divided into three risk bands, with a total DAES score ≥ 15 indicating moderate to high risk.

There is an important demand for psychometrically valid, interactive level 2 ASD screening tests that clinicians can easily learn and administer (Brewer et al., 2020). Some current Level 2 interactive screening tests require significant training and can take a long time to administer, making them difficult to integrate into clinical settings.

A strength of the DAES is that it can be promptly used in the clinical practice with “clinically referred” children who have already been assessed with the Griffiths III, to measure ASD risk at 12–36 months of age.

In addition, the DAES can be administered independently of the Griffith III in approximately 20 min as a front-line screener for ASD in 'clinically referred' toddlers aged ≤ 36 months with expressed concerns for either ASD or DD. In both instances, no additional training is required for Griffith III trained users.

### Study Limitations

The present study has certain limitations. We found a lower significant correlation between the total DAES score and the RRB CSS. This is probably related to the lack of items concerning restricted and repetitive behaviours in the DAES, derived from Griffiths III, that in turn doesn’t explore the RRB domain. The present finding is consistent with other studies on screening tools in children at risk for ASD (Rowberry et al., 2015). While it is clear that RRBs are present in young children, these studies highlighted that the RRB domain does not discriminate children with ASD from children with Development Delay/Typical Development as effectively as Social Communication symptoms when used in screening measures (Dow et al., 2017).

One more limitation is that our sample of ASD-risk children with a DA of 12–36 months, ranged in age from 18 to 48 months. Further studies are needed to extend the assessment to ASD-risk children younger than 18 months of age. On the other hand, the variability and non-linearity of the ASD phenotype in early development defines a diagnostic instability over time (“lost or later diagnosis”), with some children meeting diagnosis at follow-up, and other children no longer meeting diagnostic criteria (Landa et al., 2013, 2022). Therefore, further studies may well consider expanding the age range of the DAES, by analysing additional Griffiths III items, that may be predictive of ASD risk at older ages.

Another possible limitation of this research is that the majority of the children with ASD in the study had comorbid developmental delays in two or more domains of the Griffiths III. Nonetheless, it is essential to recognise that level 2 screeners' limitations are linked to their ability to differentiate between ASD and diverse neurodevelopmental disorders, such as DD. In this regard, studying a sample of children with ASD and DD allowed us to better identify predictive profiles that could differentiate the two conditions. This is important for aiding the process of differential diagnosis and informing individualised interventions. Further studies with larger samples of children with ASD without DD may confirm that the specific social-communicative difficulties are caused by the presence of ASD alone, rather than by the combined effects of ASD and DD.

Overall, the study findings suggest potential for a level 2 ASD screening test, but further replication in independent referral samples is necessary.

## Conclusions

Future priorities for level 2 screeners include the exploration of potentially discriminating items at the target age range while attempting to unravel the complexity of developmental trajectories in children with ASD. In this scenario, the present study aimed to develop a novel level 2 screening test for ASD based on differences in early developmental profiles on the Griffith III (DAES) that may predict an ASD-risk. The DAES includes the most predictive items for ASD-risk in children aged 18–48 months with a DA of 12–36 months, differentiating ASD-risk from DD-risk children and TD peers. The tool was developed and preliminarily validated in two phases, including two different sets of participants respectively, supporting the test effectiveness and demonstrating moderate to high correlation rates among DAES total score and ADOS-2 CSS. If the present results are replicated, the DAES has strong potential for adding to current screener efforts for early identification of children with ASD-risk enabling more informed referrals to the most appropriate diagnostic procedures and facilitating access to targeted intervention.

## Supplementary Information

Below is the link to the electronic supplementary material.

Supplementary file1 (DOCX 1247 kb)

Supplementary file2 (DOCX 213 kb)

## Abbreviations

DAES: Developmental Autism Early Screening
ASD: Autism spectrum disorder
DD: Global developmental delay
CTRL: Neurotypical control group
DA: Developmental age
CA: Chronological age
SA: Social affect
RRB: Restricted and repetitive behaviour
CSS: Calibrated severity score
PPV: Positive predictive value
NPV: Negative predictive value
