# Correction: The Developmental Autism Early Screening (DAES): a novel test for screening Autism Spectrum Disorder

**DOI:** 10.1007/s10803-024-06274-w

**Published:** 2024-03-15

**Authors:** Lara Cirnigliaro, Maria Stella Valle, Antonino Casabona, Martina Randazzo, Francesca La Bruna, Fabio Pettinato, Antonio Narzisi, Renata Rizzo, Rita Barone

**Affiliations:** 1 Child Neurology and Psychiatry Unit, Department of Clinical and Experimental Medicine, University of Catania, Policlinico Via Santa Sofia, 78, 95123 Catania, Italy; 2 Laboratory of Neuro-Biomechanics, Department of Biomedical and Biotechnological Sciences, School of Medicine, University of Catania, Catania, Italy; 3 IRCCS Stella Maris Foundation, Pisa, Italy; 4 Reseach Unit of Rare Diseases and Neurodevelopmental Disorders, Oasi Research Institute-IRCCS, Troina, Italy

**Correction: Journal of Autism and Developmental Disorders**

10.1007/s10803-023-06184-3

After the publication of [1], it has come to our attention that article’s Supplementary Information contained copyrighted material [2] that should not have been made publicly available. As a result, Table S1 in the Supplementary Information has been replaced with a new table that complements the original contents and is free from any possible copyright infringements. The title of the article has also been updated in order to emphasize the fact that this article is the result of our own independent research, and is not an additional product to the Griffiths III scales [2]. As a result, in the title and in the entire article, the name of the new screener was changed from GAES (Griffith Autism Early Screening) to DAES (Developmental Autism Early Screening). The results and conclusions of this article remain unchanged.

### Electronic Supplementary Material

Below is the link to the electronic supplementary material.

Supplementary Material 1
