# Supplementary material for: The Developmental Autism Early Screening (DAES): A Novel Test for Screening Autism Spectrum Disorder
Source: J Autism Dev Disord. 2023 Dec 18;55(1):221–36. doi: 10.1007/s10803-023-06184-3 (PMC11802666; doi:10.1007/s10803-023-06184-3)
Supplement: Supplementary file 1 — Supplementary file1 (DOCX 1247 kb) [file 10803_2023_6184_MOESM1_ESM.docx]

**Supplementary Material**

**Table S1.** Developmental Autism Early Screening (DAES): constructs, items and scoring

**DEVELOPMENTAL AUTISM EARLY SCREENING (DAES)**

| Chronological age: 18-48 months  (developmental age: 12-36 months) | |
| --- | --- |
|  | |
| CONSTRUCTS* | SCORE |
| B1.1 | **+2** |
| B1.6 | **+2** |
| B1.10 | +1 |
| B1.11 | +1 |
| B1.13 | **+2** |
| B1.14 | +1 |
| B1.16 | +1 |
| B2.2 | +1 |
| B2.3 | +1 |
| B2.9 | +1 |
| B2.10 | **+2** |
| B2.13 | **+2** |
| B2.14 | +1 |
| CONSTRUCTS** | |
| D1.3 | +1 |
| D1.4 | **+2** |
| D1.13 | +1 |
| D1.14 | **+2** |
| D1.15 | +1 |
| D1.16 | +1 |
| D1.18 | **+2** |
| D2.2 | **+2** |
| D2.3 | **+2** |
| D2.4 | +1 |
| D2.7 | +1 |
| D2.8 | **+2** |
| D2.9 | +1 |
| D2.10 | +1 |
| D2.13 | +1 |
| D2.14 | +1 |
| D2.15 | +1 |
| D3.1 | +1 |
| D3.2 | **+2** |
| D3.4 | +1 |
| D3.5 | +1 |
| D3.7 | **+2** |
| D3.8 | +1 |
|  | TOT: |

Constructs*) Listening, Attention, Communicative Intent, Preverbal Communication, Receptive Language, Expressive Language.

Constructs**) Social awareness, Self recognition, Joint attention, Expression/comprehension of emotion, Personal autonomy

Items’ administration according to Griffiths III manual (Green, E., Stroud, L., O'Connell, R., Bloomfield, S., Cronje, J., Foxcroft, C., Hurter, K., Lane, H., Marais, R., Marx, C., McAlinden, P., Paradice, R., & Venter, D. (2016). *Griffiths Scales of Child Development 3rd Ed. Part II: Administration and scoring.* Oxford, UK.: Hogrefe)

***INSTRUCTIONS FOR SCORING***

*• If the child does not pass the item (skill not achieved), assign the score + 1 / + 2 as indicated.*

*• Add up the TOT. score and assign the risk score for Autism Spectrum Disorder (Figure S2)*
