# Supplementary material for: The Developmental Autism Early Screening (DAES): A Novel Test for Screening Autism Spectrum Disorder
Source: J Autism Dev Disord. 2023 Dec 18;55(1):221–36. doi: 10.1007/s10803-023-06184-3 (PMC11802666; doi:10.1007/s10803-023-06184-3)
Supplement: Supplementary file 2 — Supplementary file2 (DOCX 213 kb) [file 10803_2023_6184_MOESM2_ESM.docx]

**Supplementary Material**

**Figure S1**


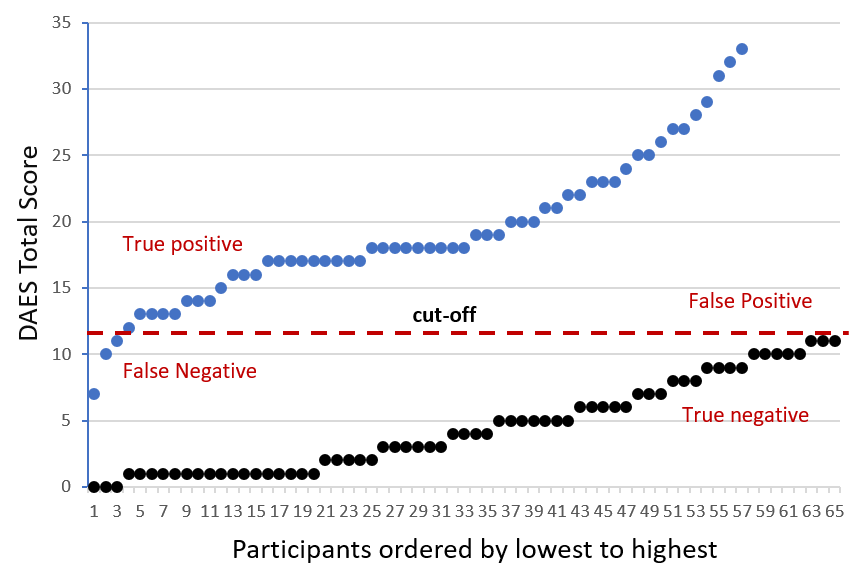
**
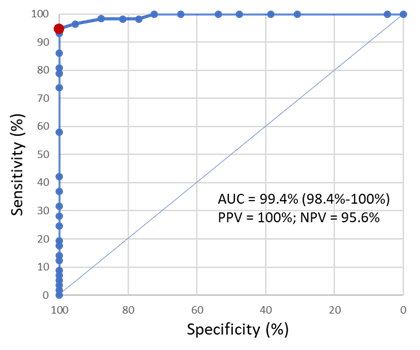
**

**(a) (b)**


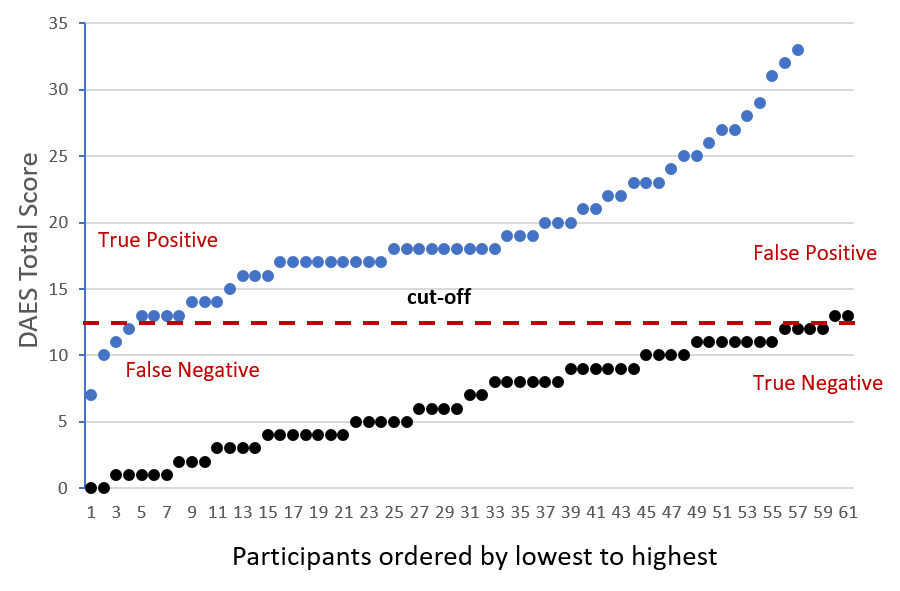
**
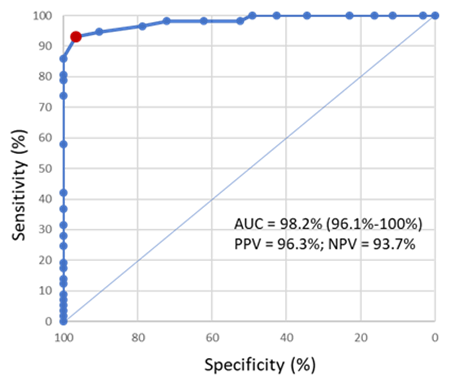
**

**(c) (d)**

**Figure S2**

**
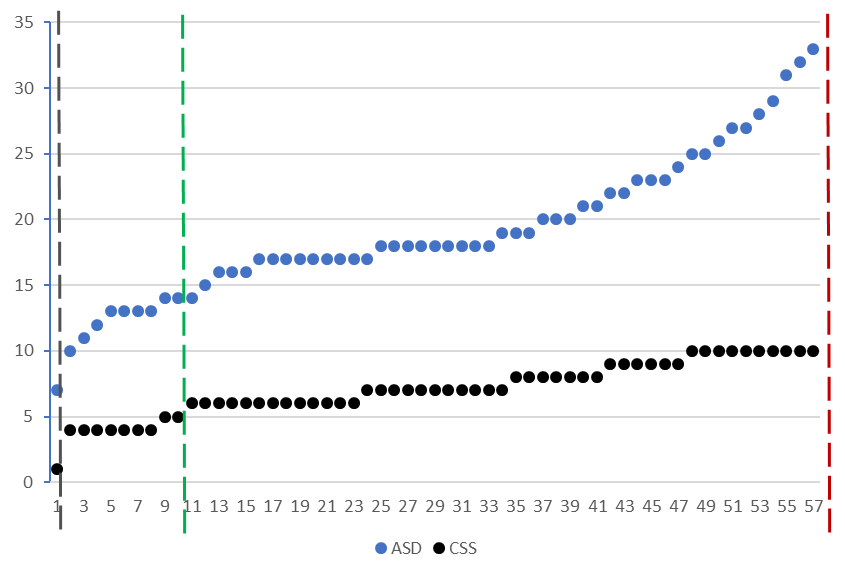
**

**
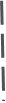
**

Little-to-no risk (CSS: 1-3, DAES total score: 1-7)

**
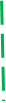
**

Mild-to-moderate risk (CSS:4-5, DAES total score: 8-14)

**
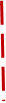
**

Moderate-to-severe risk (CSS: 6-10, DAES total score: 15-33)

| **DAES AUTISM SPECTRUM DISORDER RISK SCORE** | | |
| --- | --- | --- |
| 1-7 | 8-14 | 15-49 |
| LITTLE-TO-NO RISK | MILD-TO-MODERATE RISK | MODERATE-TO-SEVERE RISK |

**Figure Captions**

**Figure S1 (a)** ROC curve of DAES Total Score to evaluate sensitivity and specificity in separating ASD (n = 57) from CTRL (n = 65) groups. **(b)** Distribution of DAES Total Score for each participant of ASD (
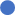
 n = 57)/ CTRL (
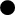
 n = 65) groups**. (c)** ROC curve of DAES total score to evaluate sensitivity and specificity in separating ASD (n=57) from GDD (n = 61) groups. **(d)** Distribution of DAES Total Score for each participant of ASD (
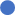
 n = 57)/GDD (
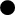
 n = 61) groups

● DAES Total Score cut-off

*AUC* Area under the curve *PPV* Positive predictive value; *NPV* Negative predictive value

**Figure S2** Risk ranges of DAES total score according to the ADOS-2 total Calibrated Severity Score

(CSS)
